# Biomarkers and *De Novo* Protein Design Can Improve Precise Amino Acid Nutrition in Broilers

**DOI:** 10.3390/ani12070935

**Published:** 2022-04-06

**Authors:** María Cambra-López, Pablo Jesús Marín-García, Clara Lledó, Alba Cerisuelo, Juan José Pascual

**Affiliations:** 1 Institute for Animal Science and Technology, Universitat Politècnica de València, Camino de Vera s/n, 46022 Valencia, Spain; claralledomorell@outlook.es (C.L.); jupascu@dca.upv.es (J.J.P.); 2 Departamento Producción y Sanidad Animal, Salud Pública y Ciencia y Tecnología de los Alimentos, Facultad de Veterinaria, Universidad Cardenal Herrera-CEU, CEU Universities, 46113 Valencia, Spain; pablo.maringarcia@uchceu.es; 3 Centro de Investigación de Tecnología Animal, Instituto Valenciano de Investigaciones Agrarias, 12400 Segorbe, Spain; cerisuelo_alb@gva.es

**Keywords:** precision livestock farming, PLF, precise feeding, poultry, ideal protein

## Abstract

**Simple Summary:**

Almost half of the protein ingested by broilers is not retained and is excreted, impairing the nitrogen utilization, health and productivity of the animals, and intensifying the environmental impact of poultry meat production. This work proposes two potential tools, combining traditional nutrition with biotechnological, metabolomics, computational and protein engineering knowledge, which can contribute to improving precise amino acid nutrition in broilers in the future: (i) the use of serum uric nitrogen content as a rapid biomarker of amino acid imbalances, and (ii) the design and modeling of *de novo* proteins that are fully digestible and fit exactly to the animal’s requirements. Both tools can open up new opportunities to form an integrated framework for precise amino acid nutrition in broilers, helping us to achieve more efficient, resilient, and sustainable production. This information can help to determine the exact ratio of amino acids that will improve the efficiency of the use of nitrogen by poultry.

**Abstract:**

Precision nutrition in broilers requires tools capable of identifying amino acid imbalances individually or in groups, as well as knowledge on how more digestible proteins can be designed for innovative feeding programs adjusted to animals’ dynamic requirements. This work proposes two potential tools, combining traditional nutrition with biotechnological, metabolomic, computational and protein engineering knowledge, which can contribute to improving the precise amino acid nutrition of broilers in the future: (i) the use of serum uric nitrogen content as a rapid biomarker of amino acid imbalances, and (ii) the design and modeling of *de novo* proteins that are fully digestible and fit exactly to the animal’s requirements. Each application is illustrated with a case study. Case study 1 demonstrates that serum uric nitrogen can be a useful rapid indicator of individual or group amino acid deficiencies or imbalances when reducing dietary protein and adjusting the valine and arginine to lysine ratios in broilers. Case study 2 describes a stepwise approach to design an ideal protein, resulting in a potential amino acid sequence and structure prototype that is ideally adjusted to the requirements of the targeted animal, and is theoretically completely digestible. Both tools can open up new opportunities to form an integrated framework for precise amino acid nutrition in broilers, helping us to achieve more efficient, resilient, and sustainable production. This information can help to determine the exact ratio of amino acids that will improve the efficiency of the use of nitrogen by poultry.

## 1. Introduction

Precision nutrition is not a new concept. It was used for the first time in poultry nutrition in 1979 in a precision feeding bioassay to measure true available amino acids in roosters [1]. Precision nutrition combines traditional nutrition with other disciplines (mathematics, computer sciences, chemistry, biochemistry, biology, immunology, molecular biology, genetics, engineering and technological sciences, amongst others) in a multi-disciplinary approach [2].

It can be defined as the practice of meeting the nutrient requirements of animals as accurately as possible in the interests of safe, high-quality and efficient production, while ensuring the lowest possible load on the environment [3]. Therefore, it aims at precisely matching animals’ nutritional requirements with adjusted feed diets, and requires a well-characterized and accurate nutrient database for each ingredient, together with properly defined animal nutrient requirements [4].

By definition, this concept is inherently linked to animal farming practices, and is key to optimizing feed efficiency for maximal economic return and minimum losses. However, despite its history of use, paradoxically, the practical implementation of precise nutrition in broiler production is not yet entirely achieved. In fast-growing broilers, nutritional requirements change quickly over time, and daily variations cannot be met with multiphase-feeding only [5,6], or by blending diets [4,7].

Nutritional requirements are commonly set for a population of similar animals (according to their age, physiological status and/or genetics, and occasionally sex). Using the population-feeding approach, individual variations within animals cannot be addressed [8], and singular needs according to nutritional status, genetics or animal health, and environmental stress-related conditions may be consequently overlooked. Managing animals individually is key in precision livestock farming [9], and can prevent over-feeding, particularly in growing pigs [8]. Nevertheless, it is questionable whether the precision management of individual birds is feasible in the poultry sector [2].

Protein over-feeding results in an increasing nitrogen (N) environmental load and ammonia emissions, and causes economic losses [10]. Birds need adjusted amino acid levels that are ideally combined (using the ideal protein concept, expressed relative to lysine [11]) to meet the requirements of each amino acid without deficit or excess. Even though broilers are one of the most efficient animals in transforming proteins into meat, compared with swine or cattle [12], their N retention is low and ranges from 57 to 60% [13]. Therefore, almost half of the protein ingested by broilers is not retained and is excreted.

Moreover, undigested protein and the metabolites from protein fermentation (ammonia, amines, p-cresol and indole [14]) can negatively affect intestinal health [15]. Undigested protein in the distal gastrointestinal tract can disrupt gut function and integrity [16], and can also be used by undesirable pathogenic bacteria [17]. Furthermore, if amino acids are available in excess or improperly balanced, they need to be catabolized in the liver. As a consequence, ammonia, which is highly toxic, is produced and must be released. The deamination of unused amino acids in the liver and the excretion of ammonia as uric acid in poultry is costly for the animal, requiring a supply of energy in the form of adenosine triphosphate (ATP)—three ATP molecules are consumed for every N molecule excreted [18]. All this seriously worsens the health and productivity of the animals and intensifies the environmental impact of poultry meat production.

Even though nowadays nutritionists use optimized feed supply and animal amino acid requirement evaluation methods (based on true ileal digestible amino acids, rather than traditional crude protein estimation or fecal total amino acids; together with modeling approaches that can assist in the process [19,20]), further research on balanced feeds, with maximal amino acid digestibility tailored for each animal’s requirements over time, is needed.

To achieve the ideal fitting of amino acid supply to animals’ dynamic requirements, precision nutrition in broilers requires tools capable of identifying deficiencies or imbalances individually or in groups, as well as knowledge on how more digestible and usable proteins can be obtained or even designed for innovative feeding programs. This new framework would help reduce the detrimental effects of protein over-feeding and inaccurate amino acid balancing in broiler diets.

This work proposes two potential tools, combining traditional nutrition with biotechnological, metabolomic, computational and protein engineering knowledge, which can contribute to improving precise amino acid nutrition in broilers in the future: (i) the use of serum uric N (SUN) content as a rapid biomarker of amino acid imbalances, and (ii) the design and modeling of *de novo* proteins that are fully digestible and fit exactly to the animal’s requirements. Each application is illustrated with a case study. The required future improvements in protein nutrition using precision nutrition tools in broilers are further discussed.

## 2. Case Study 1: Use of SUN Content as a Rapid Biomarker of Amino Acid Imbalances

### 2.1. Background

Serum uric nitrogen corresponds to the amount of N in the form of uric acid circulating in the bird’s bloodstream. Therefore, it can be used as a metabolic indicator of amino acid imbalances and deficiencies. This biomarker has become more common in the last few decades as a valid criterion to determine amino acid requirements under conditions of constant protein intake [21]. It has been successfully validated in swine [21,22,23], rabbits [24] and broilers [25].

In sows fed a diet that is adequate in the first-limiting amino acids, the concentration of plasma urea nitrogen is low because there is a decrease in protein catabolism, more efficient total N utilization, and thus a decrease in urea synthesis [21]. In rabbits, a diet with an imbalance in any essential amino acid would lead animals to catabolize the remaining amino acids, increasing the urea production in the liver, which would be released into the bloodstream, increasing plasma urea nitrogen [24]. The higher the excess of digested protein and the more limiting the affected amino acid is, the higher the plasma or SUN levels will be.

Methionine followed by lysine are the first limiting amino acids in most practical poultry diets [26,27,28], and their requirements are generally accurately estimated [29,30,31,32]. The requirements of other amino acids, however, still need further adjustment. For example, the recommendations on the valine and arginine to lysine ratios are near 0.80 [33] and 1.05 [34], respectively. Their high nutritional requirements and their relative low presence in commercial diets indicate they are relevant amino acids, which may become limiting in specific situations. Therefore, there is a need to determine their levels accurately in broiler diets to optimize both growth and N use.

This case study illustrates how SUN content can be used as a valid biomarker to detect imbalances and deficiencies in secondary limiting amino acids in broilers. The use of this biomarker is a promising tool used to verify feed formulations, monitor the ideal balancing of amino acids in broilers, and aid in adjusting amino acids to precisely match animal’s requirements over time.

A trial was conducted to determine the effects of reducing dietary protein and adjusting valine and arginine to lysine ratios in broilers. The level of SUN metabolite was used to identify potential amino acid imbalances. The relationship between SUN and performance traits was also evaluated.

### 2.2. Animals and Experimental Procedure

Three hundred and thirty-six male broilers (Ross 308) were assigned to four dietary treatments from days 14 to 35 of age. Before that, all birds were fed a commercial diet. Animals were reared in floor pens in an environmentally controlled room.

All experimental procedures used in this study were approved by Universitat Politècnica de València’s Animal Experimentation Ethics Committee, and authorized by the Valencian Conselleria de Agricultura, Medio Ambiente, Cambio Climático y Desarrollo of Spain, with the code 2017/VSC/PEA/000166.

There were seven pens per treatment (1.3 m × 1.3 m) and 12 animals/pen. Diets were formulated to meet the birds’ crude protein requirements (20%; in T1) or to be below the crude protein requirements (18%; in T2, T3 and T4) [35], combined with changes in valine (0.70 to 0.80) and arginine (0.90 to 1.05) to lysine ratios.

Diets were formulated to be isoenergetic (3000 kcal metabolizable energy/kg) and pelleted (target pellet temperature = 70 °C). The valine to lysine ratio was formulated according to current recommendations (average analyzed value of 0.81; Table 1) in T1, T2 and T4, and it was below these recommendations in T3 (0.71; Table 1). The arginine to lysine ratio was formulated according to current recommendations (average analyzed value of 1.07; Table 1) in T1, T2 and T3, and was below them in T4 (0.93; Table 1). Amino acid changes in dietary treatments were established by adding synthetic amino acids to a common basal diet based on corn, wheat and soybean meal.

Individual body weight and pen feed intake were controlled on days 14, 21, 28 and 35 of age. On day 36 of age, animals were fasted for 2 h, and blood samples were obtained 90 min after giving them access to feed. Blood samples were obtained from the wing veins in 84 animals (2115 ± 11 g body weight; 3 animals per pen; 21 animals per treatment) in 3 mL serum tubes (vacutainers). Blood samples were centrifuged (10 min, 3000× *g*) and stored frozen (−20 °C) until analyses.

The determination of SUN was performed using a commercial kit (Urea/BUN-Color, BioSystems S.A., Barcelona, Spain). Samples were firstly defrosted and tempered, and then 1 μL was pipetted into test tubes (a standard and a blank were included in each batch). Later, 1 mL of reagent A (sodium salicylate 62 mmol/L, sodium nitroprusside 3.4 mmol/L, phosphate buffer 20 mmol/L and urease 500 U/mL) was added to each sample, mixed thoroughly and incubated for 5 min at 37 °C. Subsequently, 1 mL of reactant B (sodium hypochlorite 7 mmol/L and sodium hydroxide 150 mmol/L) was added, mixed thoroughly and incubated for a further 5 min at 37 °C. Finally, the absorbance of each sample was read at 600 nm against the blank.

Individual bird SUN, final bird body weight and average daily gain (ADG) data were statistically analyzed with the GLM procedure of the SAS System Software^®^. The experimental diet (T1 to T4) was considered as the fixed effect in the model. Least square means were obtained with standard errors. Significant differences were declared at *p* ≤ 0.05.

### 2.3. Results

Table 2 shows the SUN values and productive traits (mean ± standard error of the mean) obtained for each experimental diet. The average SUN varied from 1.89 ± 0.1 to 2.26 ± 0.1 mg/dL in animals fed the tested diets. Animals fed diet T4 showed the highest SUN values (on average +18%, *p* < 0.05) compared with groups T1 to T3. These results agree with the performance data (weight and ADG), where T4 showed lower values compared with T1 and T2 (*p* < 0.05). On the other hand, the SUN concentrations were similar among treatments T1 to T3. The final weight and ADG were the highest in animals fed diet T1, medium in diet T2, and the lowest in animals fed diets T3 and T4.

### 2.4. Discussion

The serum uric N and productive traits were within the normal parameters obtained for broilers in the grower phase, and they agree with previous work [36]. Our data suggest that none of the diets offered with a low crude protein level (18% in T2 to T4) achieved the productive traits obtained with diet T1 (20% crude protein). Some authors indicate that establishing a minimum dietary crude protein content may not be necessary when proper amino acid ratios are implemented in diet formulation [37]. Moreover, research has shown that reductions in crude protein levels (below 19.5%) in broilers of similar ages can limit growth [38,39].

Animals fed diet T4 showed a significantly higher SUN compared with the rest of the animals in groups T1 to T3. Higher SUN could indicate major amino acid catabolism. Therefore, according to changes in SUN concentration, T4 would be the most unbalanced diet in terms of amino acids. These results agree with low performance data (weight and ADG) in T4.

**Figure 1:**
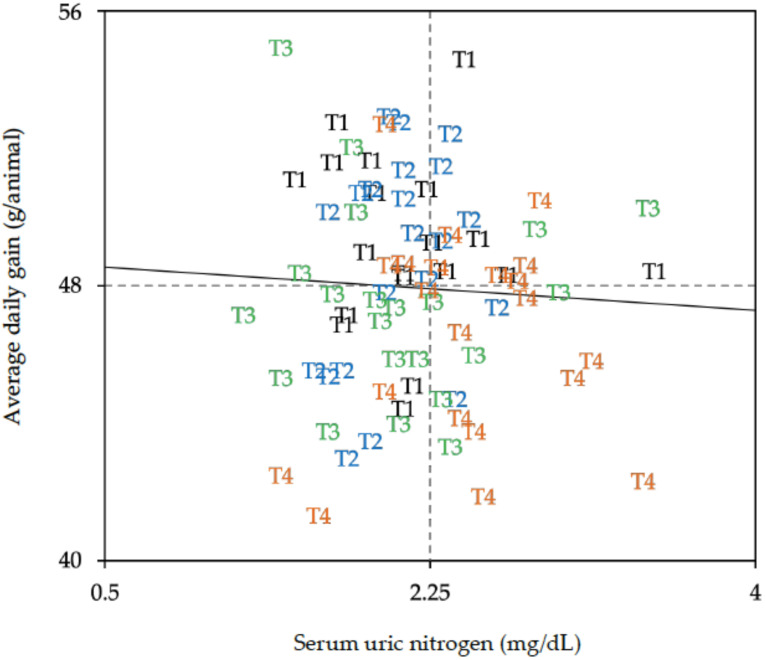
Relationship between animal’s serum uric nitrogen (SUN), when fed the different experimental diets varying in crude protein, as well as valine and arginine to lysine ratios (T1 to T4), and individual average daily weight gain during the last period of the grower phase (day 28 to 35) in broilers (*n* = 21 animals per treatment). T1: 20% crude protein content, valine/lysine ratio (0.80) and arginine/lysine ratio (1.05) formulated according to current recommendations; T2: 18% crude protein content and valine/lysine ratio (0.80) and arginine/lysine ratio (1.05) formulated according to current recommendations; T3: 18% crude protein content, below-required valine/lysine ratio (0.70) and arginine/lysine ratio (1.05) formulated according to current recommendations; T4: 18% crude protein content, below-required arginine/lysine ratio (0.92) and valine/lysine ratio (0.80) formulated according to current recommendations.

Figure 1 shows there is high individual variability in the ADG and in the SUN content amongst animals, even for those within the same dietary treatment. This figure shows that the animals fed the highest protein content (diet T1) are mostly in the upper half (high growth rate), and that in the low-growth and high-SUN quadrant, there are mainly animals fed the diet with a low arginine to lysine ratio (T4).

As regards low-protein diets, animals fed diet T2 (with valine/lysine and arginine/lysine according to current recommendations) showed similar SUN levels to those fed diet T3 (with valine/lysine ratio below current recommendations), but lower SUN levels (−16%; *p* < 0.05) than those fed diet T4 (with arginine/lysine ratio below current recommendations). These results indicate that current recommendations of valine and arginine seem to be correctly determined, as a reduction in any of them has a clear negative effect on broiler growth performance. However, only a reduction in arginine (and not valine) increased amino acids catabolism. This could imply that when lysine and methionine are well-fitted, arginine would limit the protein use of the animals more than valine (in diets with low crude protein levels). It would be interesting to verify arginine levels, since it could be the third limiting amino acid when low-protein diets based on corn, wheat and soybean meal are used in broilers. Some authors have already stated the importance of arginine when the protein level is limited [40]. In addition, positive effects have been shown when elevated levels of arginine were supplied under these conditions [41,42]. Attia et al. [43] suggested that the response to the level of amino acid addition in low-protein diets can also vary according to bird strain and age.

Although more studies are necessary to establish the potential of SUN and other biomarkers (as glutamine or glutamate in the blood) to determine amino acid imbalances in broiler diets, this study highlights the interaction of nutrition with metabolic phenotype to achieve this goal.

## 3. Case Study 2: *De Novo* Protein Design, an Example of an Ideal Protein for 21-Day-Old Broilers

### 3.1. Background

The ideal protein concept is built on the idea that birds need specific amounts and ratios of amino acids to achieve their optimal performance and maximum growth [11]. Dietary amino acid concentrations should match needs for both maintenance and muscle accretion to effectively allow for the increased synthesis of white meat [44]. However, the utilization efficiency (including digestion and metabolism) of amino acids from feed ingredients is relatively low, resulting in high N losses in excreta [13].

Moreover, with current feed formulations, the right proportion of amino acids for each animal cannot be properly achieved for all amino acids at the same time without producing an excess of some amino acids to ensure others. In addition, protein digestibility depends not only on the molecular features of the protein, but also on the action of the enzymes (i.e., proteases) involved in the digestion process. In order to allow absorption by enterocytes in the small intestinal mucosa, proteins must be broken down into dipeptides, tripeptides or free amino acids. The specificity of enzymes (stomach pepsin, pancreatic trypsin and chymotrypsin, as well as intestinal mucosal carboxypeptidases and aminopeptidases), their enzyme to substrate ratio, as well as their different ways of cleaving peptide bonds will determine the final level of protein breakdown. The structural properties of proteins (secondary structure and β-conformations) may play a major role in resistance to denaturation and gastrointestinal digestion, as well [45].

Computational and protein engineering methods could be valuable tools to help design a protein sequence and structure that meets the needs of all amino acids (without excesses or defects), and which is fully digested and metabolized in broilers. The obtained protein could be synthesized and used in the future to contribute to this goal.

In recent decades, the use of certain synthetic amino acids has allowed us to better adjust diets and reduce their protein contents. Although we are far from being able to develop completely synthetic proteins that can cover these needs in a profitable way, the exponential development of biotechnology could get us close to that reality in the coming years. Therefore, knowledge of how to develop this technology is necessary.

This case study illustrates how *de novo* protein design can contribute to this aim. It describes a novel stepwise modeling approach to designing an ideal protein that can be completely digestible and usable in broilers from 0 to 21 days of age. It is an example of how precision nutrition strategies combining traditional nutrition with biotechnological, computational and protein engineering approaches can contribute to addressing precision poultry nutrition challenges in the future. The boundary conditions set in this case scenario include: (i) a primary protein sequence design containing the minimal amino acid quantities that can be fully digested by enzymes from the avian digestive system, and (ii) modeling the secondary and tertiary conformations of already-designed polypeptides.

### 3.2. Experimental Procedure

To this end, firstly, a literature review was conducted to define the requirements of amino acids for 21-day-old broilers, as regards the amino acid composition and ideal protein profile. Secondly, the protein digestion dynamics and functioning of the digestive system at the enzymatic level of chickens of that age were also reviewed. From these data, potential primary polypeptide sequences were designed. We chose the shortest protein sequence that fulfilled the following criteria: (i) fully meeting broiler requirements of all amino acids, while (ii) optimizing digestive enzyme functioning. Finally, we predicted its secondary and tertiary structure and its physicochemical properties using computational methods.

The *de novo* design of a protein that fully meets the requirements of broilers should be based on the net amino acid requirement (maintenance plus growth requirements) at each stage of the animal’s life. Although there have been some attempts to obtain this information [46], it is not yet available. For this reason, the present work was based on the closest estimates of net requirements, which correspond to the true ileal digestible amino acids (i.e., corrected for basal and specific endogenous amino acid secretions).

The amino acid requirements are outlined in Table 3, based on the ideal amino acid profiles for broilers from 0 to 21 days with respect to lysine, as proposed by the North American Texas AM University [47]. These data were selected after comparing them with the available literature and recognized international nutritional guidelines for broilers (Canadian NRC [48], Spanish FEDNA [49], Dutch CVB [50] and Brazilian Tables for poultry and swine [51]). Wu’s [47] dataset, containing a total of 108 amino acids (Table 3), was chosen to construct the “minimal ideal protein”, because these recommendations are not far from the current recommendations for most of the amino acids provided by FEDNA [49] and NRC [48]. Moreover, it was the only one that provided recommendations for the 20 amino acids, and it was derived from true ileal digestible amino acid contents, accounting for the proportions of amino acids in the whole bodies of broilers.

From the 108 amino acids described in Table 3, an initial protein sequence was generated using RandSeq (from the ExPASy online portal, SIB Bioinformatics Resource Portal). This online tool is frequently used to build randomly scrambled peptide libraries from a specific amino acid composition [52,53].

Using the random sequence obtained using RandSeq, several primary structures were designed using the Peptide Cutter software’s information (ExPASy Bioinformatics Portal, Swiss Institute of Bioinformatics). The Peptide Cutter software considers the performance (activity and substrate specificity) of avian digestion enzymes in the sequence and maximizes the number of cleavages by enzymes in the linear polypeptide chain. The choice of enzymes was based on the work of Recoules et al. [54], using in vivo data on the digestion of plant proteins in broilers. Pepsin, trypsin, chymotrypsin, elastase, prolidase, carboxypeptidase A and B and aminopeptidase were studied.

The final sequences obtained were subjected to a manual refinement step to increase their potential digestibility. In other words, we increased the number of free amino acids in the final sequence by adding extra specific amino acids that would break the remaining dipeptides. Such extra amino acids were chosen following two criteria: being the target amino acid of various avian digestive enzymes and having been rounded down in the proposed minimum ideal protein initial sequence.

After this step, its secondary and tertiary structures were predicted using two online servers: i-TASSER (iterative threading assembly refinement, a hierarchical protocol for the structural and functional prediction of amino acid sequences [55]) and QUARK (based on ab initio folding, the construction of protein structures by fragment assembly from unrelated proteins [56]). Both software were used to predict the folding of sequences.

### 3.3. Results

**Figure 2:**
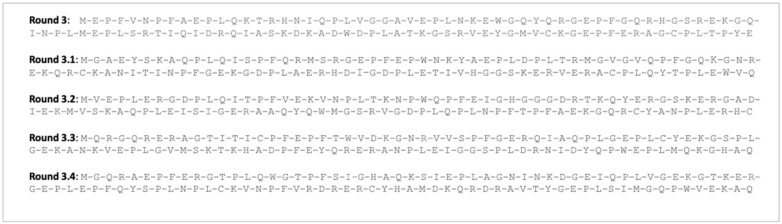
Original 108-amino acid sequence (Round 3) and refined sequence with 112 amino acids modeled for complete digestion (Round 3.1, Round 3.2, Round 3.3 and Round 3.4). One-letter amino acid code: A—alanine, C—cysteine, D—aspartic acid, E—glutamic acid, F—phenylalanine, G—glycine, H—histidine, I—isoleucine, K—lysine, L—leucine, M—methionine, N—asparagine, P—proline, Q—glutamine, R—arginine, S—serine, T—threonine, V—valine, W—tryptophan, Y—tyrosine.

Figure 2 shows the optimal initial primary amino acid sequence derived from the information in Table 3, and the protein digestion dynamics data from enzyme affinities (Round 3, Figure 1). The round 3 sequence was the most digestible sequence based on the action of the chicken digestive enzymes, because it led to a high number of free amino acids after digestion—only four dipeptides and 100 free-amino acids (7.4 and 92.6% of the total amino acids in the sequence, respectively).

Rounds 3.1 to 3.4 (Figure 2) were extra sequences generated from the original sequence (Round 3) during manual refinement. These extra sequences (Rounds 3.1 to 3.4) were designed to break the four remaining dipeptides following complete digestion. To this end, four extra amino acids were included in the composition (a total of 112 amino acids), with the following considerations: (i) prioritizing those amino acids that were a frequent target for digestive enzymes in chickens (arginine, isoleucine, leucine, lysine, phenylalanine, tryptophan and tyrosine); (ii) promoting isoleucine addition, given that it is rounded down to avoid shortage; (iii) giving special attention to lysine, due to its roles as the first limiting and the reference amino acid, making it worthwhile to ensure its minimal requirement is met; (iv) adding arginine and tryptophan, due to their relevance as first limiting amino acids; (v) avoiding cysteine (due to the risk of disulphide bridges), which reduces digestive enzymes’ efficiency. The addition of the four extra amino acids resulted in increases in the amounts of isoleucine by 25.0%, lysine by 16.7%, arginine by 14.3% and tryptophan by 100.0%.

**Figure 3:**
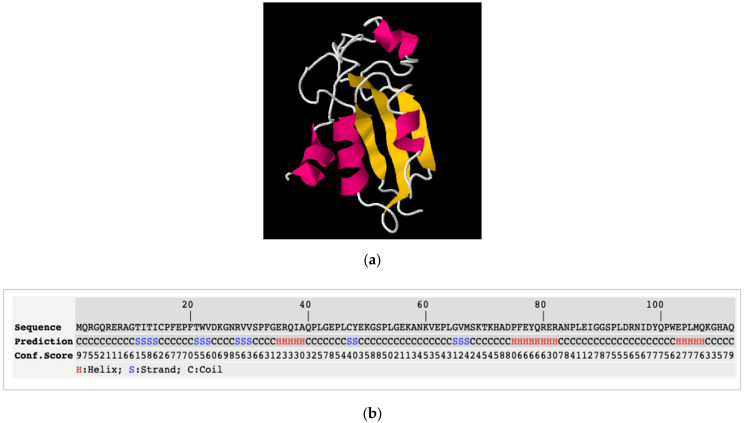
Predicted secondary and tertiary structure of sequence Round 3.3 by I-TASSER. (**a**) Three-dimensional tertiary structure cartoon model. In pink α-helices, in yellow β-sheets and in white coil regions. (**b**) Secondary predicted structure. H: α-helices; S: β-sheets; C: coil regions based on *in silico* digestion of initial designed sequences for primary structure. One letter amino acid code: A—alanine, C—cysteine, D—aspartic acid, E—glutamic acid, F—phenylalanine, G—glycine, H—histidine, I—isoleucine, K—lysine, L—leucine, M—methionine, N—asparagine, P—proline, Q—glutamine, R—arginine, S—serine, T—threonine, V—valine, W—tryptophan, Y—tyrosine.

Finally, secondary and tertiary protein structures were determined and tested to evaluate the quality and reliability criteria of the structural models obtained with the different protein sequences, using I-TASSER and QUARK software. Figure 3 presents the Round 3.3 sequence, which was the most reliable and highest-quality model, as indicated by its C-score (accuracy ranging from −5 to 2, increasing with high confidence) and TM-score (similarity to native structures, with a TM-score > 0.5, similar topology, and <0.3 random similarity). The C-score and TM-score values in Round 3.3 were higher than those of the other sequences determined using I-TASSER (on average, C-score −1.08 vs. −3.45, and TM-score 0.58 vs. 0.34). Theoretically, this implies higher reliability when predicting the actual 3D protein conformation. A complete description of all sequence quality and reliability criteria has been given by Lledó [57].

**Figure 4:**
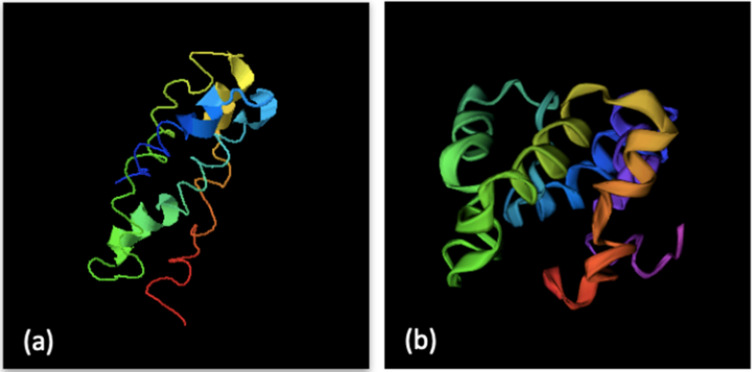
Protein 3D structure model of Round 3.1 using (**a**) the I-TASSER model and (**b**) the QUARK model.

Figure 3 shows the overall sequence covered by α-helices, β-sheets and random coil regions, derived from the i-TASSER models of Round 3.3. The secondary structure of the protein simulated in Round 3.3 showed the highest amount of β structures (13%) and the least α-helices (16%) among all candidates, and therefore it may be the least digestible protein. Following the same criteria, the secondary structure of the protein modelled in Round 3.1 (Figure 4) contained the lowest percentage of β-sheets (2%), and simultaneously the highest number of α-helices (41%), among all the models. Regarding the number of coil regions, as their conformational prediction is more intricate, these regions could display more unexpected folds. Therefore, defined structures and α-helices are preferable. The structure of the protein simulated in Round 3.1 presented one of the lowest percentages of coil regions (on average, 57% vs. 70%).

Therefore, based on the structural motifs, the protein with the highest number of α-helices and the lowest number of β-sheet was that in Round 3.1. Moreover, it was ranked second in terms of reliability on the basis of its C-score and TM-score, showing a more acceptable quality level (−1.99 and 0.48, respectively) with respect to Round 3.3 (−1.08 and 0.58, respectively). Figure 4 shows the structural properties of the Round 3.1 protein modeled using different software.

### 3.4. Discussion

The efficiency of the use of ingested dietary protein by broilers depends on the digestibility and balance of the amino acid content relative to the animal’s requirements. Increasing the crude protein content has been proven to entail negative effects in chicken health, and in environmental and production costs [58]. On the other hand, low-crude protein level diets with the addition of crystalline amino acid do not constitute a complete solution, because this can reduce chicken growth performance.

The “perfect” diet, in terms of protein supply, could involve feeds with low level inclusions of highly purified and digestible proteins. This work has outlined a novel approach, combining the structural digestibility, quality, and reliability criteria of the predicted model, resulting in a valid protein design that will help to achieve this goal.

The resulting protein model had a minimal size, with 112 amino acids. This size seems adequate to produce the ideal protein, as it is the closest to the ideal amino acid profile of broilers. Low-molecular weight proteins are more easily produced and secreted, and are less likely to interact with the host’s metabolism. This could be advantageous in terms of future biological synthesis and industrial production.

In terms of protein digestibility and solubility, it is crucial to consider the occurrence of two main structural patterns: α-helices and β-sheets. Carbonaro et al. [45] studied in vitro the structure–digestibility relationship of different proteins of animal and plant origin, and quantified the different structural motifs. Their results, consistent with other experiments [59,60], showed a reduction in hydrolysis degree that was inversely proportional to the number of β-sheets. The main explanation for this lies in the hydrophobic character of these structures, which promotes aggregation and protein–protein interactions.

Obtaining an accurate model for secondary and tertiary structures is essential, since these structures are closely related to proteins’ physical (solubility, aggregation and secretory ability) and functional characteristics. Protein solubility is very relevant to production processes, as it mainly affects cell excretion and downstream recovery processes, given that it is directly related to the aggregation phenomena. Moreover, secondary structural motifs can also affect peptide solubility. Solubility has been shown to increase with the ratio of α-helices to β-sheets in in vitro experiments [61]. 

Besides this, secondary and tertiary structural modeling can give clues about protein functional features and behaviors. The potential of the protein prototype is also strongly determined by the existence of protein templates in protein data banks with significant sequence similarity to the problem sequence. In other words, sequences without equivalents in protein data banks will be more difficult to model, and the result will be based on less evidence, leading to less reliable results overall. However, a unique protein, whose structure greatly differs from any known homologous proteins, could be advantageous. Firstly, it could decrease the mimic phenomenon, which could lead to problems in the host used for future production (bacteria, yeast, or any other chosen organism). Secondly, it is more probable that, if it does not belong to any protein family, it will not have any relevant function itself.

Through the procedure followed in this work, we obtained a prototype that meets most of the conditions that a synthetic protein should have, such as being completely digestible, not generating an excess supply of amino acids (since it is ideally adjusted to the requirements of the targeted animal), and therefore coming as close as possible to the concept of an ideal protein. In any case, more studies are necessary to improve the definition of this protein prototype that will consider the characteristics of the potential hosts, before carrying out pilot tests aimed at biosynthesis. Current protein yields using plant or microbial fermentation synthesis are still insufficient to produce a fully viable synthetic protein source for poultry. However, the efficiency of these processes is rapidly improving, and new developments in bioprocesses are emerging [62], as are innovative procedures, such as cell-free protein synthesis [63]. The use of these novel protein synthesis methods could contribute to making this procedure a reality in the near future.

## 4. Future Precision Nutrition Needs to Improve Protein Nutrition in Broilers

**Figure 5:**
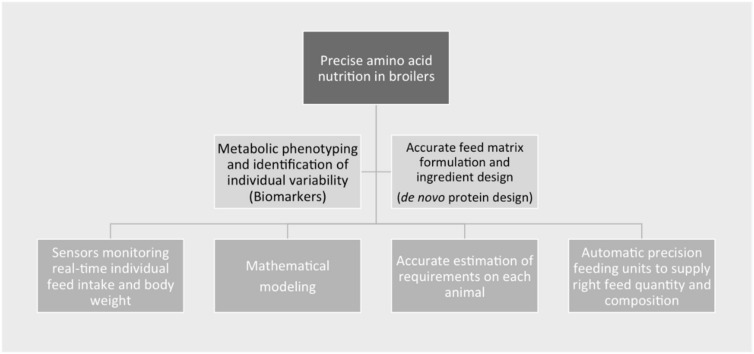
Integrated framework scheme for precise amino acid nutrition in broiler farming, combining nutritional strategies and feeding technologies.

Precision nutrition is an essential part of precision livestock farming, as both pursue a common goal: enhancing farm profitability, efficiency, and sustainability [64]. In our work, potential tools for the development of future precision nutrition strategies that will improve amino acid utilization in broilers are presented and discussed. The tools presented herein include biotechnological, metabolomic, computational and protein engineering approaches, and they are summarized in Figure 5, focusing on metabolic phenotyping and the identification of individual variability using biomarkers (Case study 1), and accurate feed matrix formulation and ingredient design (partial or complete) through *de novo* protein development (Case study 2).

The precision nutrition strategies presented in this work differ from precision feeding technologies. The latter relies on monitoring the amount and composition of the feed delivered to broilers, individually or in groups, as well as their feeding behavior and intake [4]. Precision feeding technologies require the development of sensors and automatic measuring devices, as well as sophisticated feeding units capable of providing the required amount and composition of feeds according to specific production objectives, based on growth models derived from computational methods, i.e., customized diets [65].

Therefore, the on-farm application of precision feeding technologies in broilers is still limited, and there are few examples of the practical use of such technologies. For instance, in poultry, individual monitoring is complex, and it has only been implemented in broiler breeders using feeding cubicles, based on using bird radio frequency identification (RFID), weighing in the cubicle, and a target growth curve updated in real-time [66]. Although RFID systems can accurately detect and record the feeding behaviors of individual broilers in group settings automatically [67], their field implementation is challenging, costly and complex. Furthermore, the estimation of individual nutritional requirements in real-time is not entirely feasible, and must be based on mathematical models and theoretical estimations.

In contrast, the precision nutrition strategies addressed in this work are designed to equal the dietary nutrient supply to the nutrient requirements of the animals, particularly focusing on amino acids. There is still a need to adjust the exact combination of indispensable amino acids so that they exactly match the animals’ requirements for protein accretion and maintenance, with no deficiencies and no excesses using the ideal protein profile [11]; but also to come up with valid tools that can give real feedback using animal-based biomarkers. These strategies are necessary prerequisites that must be implemented in future automated and tailored feeding technologies. In fact, as Moss et al. [4] stated, the implementation of precision nutrition relies on the ability of the poultry industry to employ precision feeding within its operations, and therefore, precise nutrition strategies must be combined with precise feeding technologies.

The tools addressed in the present case studies are therefore key to formulating an integrated framework for precise amino acid nutrition in broilers. Figure 5 illustrates how biomarkers and *de novo* protein design can fit into this scheme, as indispensable to the precise livestock farming matrix that combines feeding technologies (including sensors and automatic feeding units), modeling at individual or group levels, and nutritional strategies in an interdisciplinary approach.

## 5. Conclusions

Specific biomarkers, such as SUN, can be useful as rapid indicators of individual or group amino acid deficiencies or imbalances. Their practical implementation on-farm, as well as in nutritional research studies, could contribute to the achievement of both precise diet formulation and the determination of nutrient requirements in broilers. These types of biomarkers could be a suitable tool for closing the gap between models and farm conditions, as they could be used as an indicator of how the modelled theoretical or estimated requirements need to be adjusted in farm conditions. Broilers in different farm settings can be exposed to stressors (environmental, social and nutritional, amongst others). Individual metabolic phenotyping using biomarkers could contribute to optimizing nutrient use, reducing safety margins, and preventing nutrient over-feeding.

Furthermore, the possibility of designing a specific protein that can minimize N losses, and maximize its digestibility and metabolic use, was discussed in this work. We developed a stepwise approach to designing an ideal protein that can be completely digestible and usable in broilers from 0 to 21 days of age. The procedure presented in this work is promising for the initial design of synthetic oligopeptides that could be used in a similar way to current synthetic amino acids, and ultimately for the synthesis of proteins that meet the needs of animals exactly. Its application could help us to precisely match nutrient supply with the nutrient requirements of animals without excesses, thus minimizing losses.

In conclusion, both tools presented herein can open up new opportunities in the context of future broiler nutrition and precision farming, helping us to achieve more efficient, resilient, and sustainable production. This information can help us to determine the exact ratio of amino acids that will improve the efficiency of the use of N by poultry.

## Figures and Tables

**Table 1:** Analyzed levels of crude protein, valine and arginine, valine to lysine ratio and arginine to lysine ratio in the different dietary treatments (T1 to T4).

	Crude Protein (%)	Valine (%)	Arginine (%)	Valine to Lysine	Arginine to Lysine
T1	20.00	0.896	1.228	0.815	1.116
T2	18.13	0.873	1.136	0.806	1.049
T3	18.07	0.770	1.136	0.711	1.049
T4	17.88	0.874	1.004	0.807	0.927

Different levels of valine and arginine were achieved by adding synthetic L-valine and L-arginine. T1: 20% crude protein content, valine/lysine ratio (0.80) and arginine/lysine ratio (1.05) formulated according to current recommendations; T2: 18% crude protein content and valine/lysine ratio (0.80) and arginine/lysine ratio (1.05) formulated according to current recommendations; T3: 18% crude protein content, below-required valine/lysine ratio (0.70) and arginine/lysine ratio (1.05) formulated according to current recommendations; T4: 18% crude protein content, below-required arginine/lysine ratio (0.92) and valine/lysine ratio (0.80) formulated according to current recommendations.

**Table 2:** Average serum uric nitrogen (SUN) and productive traits (± standard error of the mean) obtained in each experimental diet (T1 to T4) during the grower phase (from 14 to 35 days of age).

	T1	T2	T3	T4	*p*-Value
SUN (mg/dL) ^1^	1.96 ± 0.1 ^a^	1.89 ± 0.1 ^a^	1.90 ± 0.1 ^a^	2.26 ± 0.1 ^b^	0.001
Final weight (g)	2188 ± 18.4 ^c^	2143 ± 18.4 ^b^	2073 ± 18.4 ^a^	2055 ± 18.4 ^a^	0.028
Average daily gain (g/d)	109.3 ± 1.62 ^c^	105.7 ± 1.62 ^b^	100.8 ± 1.62 ^a^	101.7 ± 1.62 ^a^	0.043

^a, b, c^: means in the same row with no common superscripts differ significantly (*p* < 0.05). ^1^: Obtained from 36-day-old broilers. T1: 20% crude protein content, valine/lysine ratio (0.80) and arginine/lysine ratio (1.05) formulated according to current recommendations; T2: 18% crude protein content and valine/lysine ratio (0.80) and arginine/lysine ratio (1.05) formulated according to current recommendations; T3: 18% crude protein content, below-required valine/lysine ratio (0.70) and arginine/lysine ratio (1.05) formulated according to current recommendations; T4: 18% crude protein content, below-required arginine/lysine ratio (0.92) and valine/lysine ratio (0.80) formulated according to current recommendations.

**Table 3:** Amino acid requirements (expressed relative to lysine, lysine = 100) for chickens from 0 to 21 days used for protein modeling.

Amino Acid	Mw (g/mol)	Amino Acid/Lysine ^1^	Molecules in the Sequence ^2^
Alanine	89.09	102	6
Arginine	174.2	105	7
Asparagine	132.1	56	4
Aspartate	133.1	66	4
Cystein	121.2	32	2
Glutamate	147.1	178	11
Glutamine	146.2	128	8
Glycine	75.1	176	11
Histidine	155.2	35	2
Isoleucine	131.2	67	4
Leucine	131.2	109	7
Lysine	146.2	100	6
Methionine	149.2	40	3
Phenylalanine	165.2	60	4
Proline	115.1	184	12
Serine	105.1	69	4
Threonine	119.1	67	4
Tryptophan	204.2	16	1
Tyrosine	181.2	45	3
Valine	117.2	77	5
		Total number of amino acids	108
		Mw (g/mol) ^3^	12095.1

Mw: molecular weight. ^1^: Calculated from true ileal digestibility data from Wu [47]. ^2^: As the amino acid that is required in the lowest relative proportion with respect to lysine is tryptophan at 0.16, the number of molecules in the sequence was calculated to have at least one representative of tryptophan in the protein sequence. The rest of the amino acids were proportionally calculated according to this value, as (amino acid/lysine)/0.16. ^3^: Calculated as the sum of the individual amino acid’s molecular weight × the number of molecules in the sequence, minus the molecular weight of the 107 peptide bonds (condensation reactions; 18 g/mol per bond) needed to generate the 108-amino acid sequence.

## Data Availability

Data is contained within the article.
